# NANO.PTML model for read-across prediction of nanosystems in neurosciences. computational model and experimental case of study

**DOI:** 10.1186/s12951-024-02660-9

**Published:** 2024-07-23

**Authors:** Shan He, Karam Nader, Julen Segura Abarrategi, Harbil Bediaga, Deyani Nocedo-Mena, Estefania Ascencio, Gerardo M. Casanola-Martin, Idoia Castellanos-Rubio, Maite Insausti, Bakhtiyor Rasulev, Sonia Arrasate, Humberto González-Díaz

**Affiliations:** 1 Department of Coatings and Polymeric Materials, North Dakota State University, Fargo, ND 58108 USA; 2 Department of Organic and Inorganic Chemistry, University of Basque Country UPV/EHU, Leioa, 48940 Spain; 3 IKERDATA S.L., ZITEK, UPV/EHU, Rectorate Building, nº 6, Leioa, 48940 Greater Bilbao, Basque Country, Spain; 4 Faculty of Physical Mathematical Sciences, Autonomous University of Nuevo León, San Nicolás de los Garza, 66455 Nuevo León, México; 5 BCMaterials, Basque Center for Materials, Applications and Nanostructures, Leioa, 48940 Spain; 6 BIOFISIKA: Basque Center for Biophysics CSIC, University of The Basque Country (UPV/EHU), Barrio Sarriena s/n, Leioa, 48940 Bizkaia, Basque Country, Spain; 7 IKERBASQUE, Basque Foundation for Science, Bilbao, 48011 Biscay, Spain

**Keywords:** Neurodegenerative disease, Nanoparticle, Drug carrier, Information fusion, Machine learning

## Abstract

Neurodegenerative diseases involve progressive neuronal death. Traditional treatments often struggle due to solubility, bioavailability, and crossing the Blood-Brain Barrier (BBB). Nanoparticles (NPs) in biomedical field are garnering growing attention as neurodegenerative disease drugs (NDDs) carrier to the central nervous system. Here, we introduced computational and experimental analysis. In the computational study, a specific IFPTML technique was used, which combined Information Fusion (IF) + Perturbation Theory (PT) + Machine Learning (ML) to select the most promising Nanoparticle Neuronal Disease Drug Delivery (N2D3) systems. For the application of IFPTML model in the nanoscience, NANO.PTML is used. IF-process was carried out between 4403 NDDs assays and 260 cytotoxicity NP assays conducting a dataset of 500,000 cases. The optimal IFPTML was the Decision Tree (DT) algorithm which shown satisfactory performance with specificity values of 96.4% and 96.2%, and sensitivity values of 79.3% and 75.7% in the training (375k/75%) and validation (125k/25%) set. Moreover, the DT model obtained Area Under Receiver Operating Characteristic (AUROC) scores of 0.97 and 0.96 in the training and validation series, highlighting its effectiveness in classification tasks. In the experimental part, two samples of NPs (Fe_3_O_4__A and Fe_3_O_4__B) were synthesized by thermal decomposition of an iron(III) oleate (FeOl) precursor and structurally characterized by different methods. Additionally, in order to make the as-synthesized hydrophobic NPs (Fe_3_O_4__A and Fe_3_O_4__B) soluble in water the amphiphilic CTAB (Cetyl Trimethyl Ammonium Bromide) molecule was employed. Therefore, to conduct a study with a wider range of NP system variants, an experimental illustrative simulation experiment was performed using the IFPTML-DT model. For this, a set of 500,000 prediction dataset was created. The outcome of this experiment highlighted certain NANO.PTML systems as promising candidates for further investigation. The NANO.PTML approach holds potential to accelerate experimental investigations and offer initial insights into various NP and NDDs compounds, serving as an efficient alternative to time-consuming trial-and-error procedures.

## Introduction

Neurodegenerative Diseases (NDs) constitute a diverse set of conditions marked by the gradual deterioration and loss of neurons in various regions of the nervous system. These diseases pose a significant challenge to global health because their incidence is increasing. With the expansion of the aging population, the World Health Organization anticipates a threefold increase worldwide in the number of individuals affected by neurodegenerative disorders over the coming three decades. Although the precise mechanisms driving NDs are not fully elucidated, researchers suggest a multifaceted interplay involving genetic, epigenetic, and environmental factors. Presently, there are no established treatments capable of slowing, halting, or preventing the progression of any NDs [1, 2]. For example, diseases like Alzheimer´s and Parkinson´s, which have been recognized for over a century, continue to lack a cure [3–5]. Some promising lines of research for the treatment of neurodegenerative disorders are: gene therapy [6], development of neuroprotective mimetic peptides [7], repurposing (or reevaluation) of known drugs [8], among others [9].

One challenge is the interaction between NPs and components of the immune system. Over the past ten years, research has demonstrated that although NP can be toxic, advances in nanotechnology have enabled the modification of these materials. These modifications can either prevent interaction with the immune system or specifically target it. When nanoparticles are used for medical purposes that do not aim to activate or suppress the immune system, it is beneficial to avoid any immune system interaction [10]. For instance, NPs can be engineered by coating them with poly(ethylene glycol) (PEG) or other polymers, creating a hydrophilic layer that conceals them from the immune system’s detection [11]. Another challenge to be addressed in the treatment of neurodegenerative disorders lies in the passage of therapeutic agents through the Blood-Brain Barrier (BBB) to reach the Central Nervous System (CNS). To overcome these obstacles, research efforts are directed towards both the development of new drugs and the exploration of innovative drug delivery methods, including targeted nanocarriers [12]. Some of these approaches are: nanobodies [13], nano-antibodies, nano-metal particles (gold, silver, iron oxide) [14] and lipid nanoparticles (nanoliposomes) [15]. These nano-approaches applied to drug R&D as innovative delivery systems for NDs face inherent challenges. Therefore, we find ourselves with arduous experimental work associated with high costs, low stability profiles, short useful lives, and inconsistency between and within production batches [16].

In this sense, Machine Learning (ML) techniques can be useful for analyzing, predicting, and selecting the optimal delivery nano-system to treat neurodegenerative diseases (Nanoparticle Neuronal Disease Drug Delivery systems, in the future “N2D3 systems”). ML has been successfully used for the prediction of biomedical properties of NPs of medical interest. These studies include the influence of particle physicochemical properties on cellular uptake, cytotoxicity, molecular loading, and molecular release, as well as manufacturing properties such as NP size and polydispersity [17, 18]. In the efforts to design new N2D3 systems, a ML algorithm needs to analyze multiple output properties (IC_50_, K_i_, etc.) of a broad range of N2D3 systems with different transported substances (drugs), nanocarriers, coatings, etc., under various conditions such as cell lines and organisms (labels) [19]. On the other hand, Gajewicz et al. [20] have recently discussed the lack and/or dispersion (different sources of information) of nanotoxicity data with special emphasis on the low variety of drugs transported by the current N2D3 systems in contrast to the high number of free drugs assays [21–25]. Consequently, in order face N2D3 systems design problem a ML should be multi-output (able to predict multiple outputs), read-across species (able to infer properties for different species), multi-label (able to consider multiple cell lines, etc.), and able to consider multiple sources of information at the same time. With this purpose our group introduced the Information Fusion (IF) + Perturbation-Theory (PT) + Machine Learning (ML) algorithm. IFPTML gets information from different sources (Drugs assays, NP assays, Proteomics, Metabolic networks, etc.), and carry out an IF process, later uses PT operators to quantify all the variability of the data, and last use ML algorithms to seek a predictive model and predict new N2D3 systems. In the case of specific applications to Nanoscience the algorithm has been called as the NANO.PTML algorithm. NANO.PTML algorithm have been applied successfully before to different types of NP systems [26–29].

In this paper, firstly we are going to use NANO.PTML algorithm to find a new ML model able to predict new N2D3 systems. Furthermore, in order to illustrate the applicability of the NANO.PTML model in practice we reported an additional computational-experimental case of study. In this case of study, firstly we carried out the synthesis and characterization of two new NPs with potential application in the development of N2D3 systems. Next, we used the NANO.PTML model to carry out a simulation of the outcomes for 500,000 different assays of N2D3 systems based on the two NPs reported. These predictions involve different combinations of up to 123 drugs, 53 cell lines, 16 NP coats, 5 NP core types, 5 NP shapes. The outcome of this experiment serves as guidance for the identification of promising N2D3 systems and gaining insights into their behaviors across different cell lines, coating agents, among others, which could offer valuable guidance for future studies. NANO.PTML model predictions and its experimental validation could offer a promising alternative to traditional trial-and-error methods and pave the way for more efficient N2D3 systems for neurodegenerative diseases.

## Materials and methods

### Experimental methods

#### Materials

The products, iron(III) chloride, 1-octadecene, oleic acid, dibenzyl ether, Chloroform and Cetyl Trimethyl Ammonium Bromide (CTAB) were purchased in Sigma-Aldrich. Sodium oleate, ethanol, hexane and tetrahydrofuran were purchased in TCI, PanReac, Honeywell and Emplura, respectively.

#### Experimental characterization

X-ray diffraction (DRX) patterns of Fe_3_O_4_ hydrophobic NPs were performed using a PANalytical X’Pert PRO diffractometer equipped with a copper anode (operated at 40 kV and 40 mA), diffracted beam monochromator and PIXcel detector. Scans were collected in the 10 − 90° 2θ range with a step size of 0.02° and scan step speed of 1.25 s. The amount of organic matter in the Fe_3_O_4_ hydrophobic NPs was determined via thermogravimetric measurements (TGA), performed in a NETZSCH STA 449 C thermogravimetric analyser, by heating 10 mg of dry samples at 10 °C/min in Ar atmosphere. Dynamic Light Scattering (DLS) and Zeta potential (ζ) measurements of the NPs functionalized with CTAB were performed in a Zetasizer Nano-ZS (Malvern Instruments). The measurements were carried out at 25 °C after an equilibrium time of 1 min for 0.05 mg·mL^− 1^ Fe_3_O_4_@CTAB aqueous dispersions. For each sample, 10 runs of 10 s were performed with three repetitions. A Phillips CM200 Transmission Electron Microscopy (TEM) with an accelerating voltage of 200 kV and a point resolution of 0.235 nm was used to analyse the morphology of the samples. Magnetic measurements as a function of the magnetic field M(H) at Room Temperature (RT) were obtained in a Vibrating-Sample Magnetometer (VSM) by measuring the magnetization of the dried hydrophobic nanoparticles and normalizing the magnetization value per unit mass of inorganic matter.

#### Preparation of Fe_3_O_4_nanoparticles

Two different Fe_3_O_4_ nanoparticles (NPs) were synthesized by thermal decomposition of an iron(III) oleate (FeOl) precursor which was previously prepared from iron(III) chloride and sodium oleate mixed in a mixture of solvents (hexane, ethanol and distilled water). The synthesis process of both the FeOl precursor and the Fe_3_O_4_ NPs was formerly analyzed and optimized throughout different works [30–32].

For this work two different samples composed of NPs of similar average dimension (≈ 20 nm) but different morphology (cuboctahedral and octahedral) have been prepared (samples Fe_3_O_4__A and Fe_3_O_4__B). Sample Fe_3_O_4__B was prepared by mixing 10 mmol of the previously prepared FeOl precursor with 20 mL of 1-octadecene, 10 mL of dibenzyl ether and 6.4 mL of oleic acid and heating the mixture until reflux (around 320 ºC). The resulting hydrophobic NPs of Fe_3_O_4_ coated with oleic acid were washed by centrifugation 3 times (at 9500 rpm) with ethanol and tetrahydrofuran and, finally, they were collected in chloroform and stocked in the fridge 4 ºC. Fe_3_O_4__A was similarly prepared, but in this case the synthesis process was scaled to double to analyze the effect of this synthetic parameter in the features of the NPs.

**Fig. 1:**
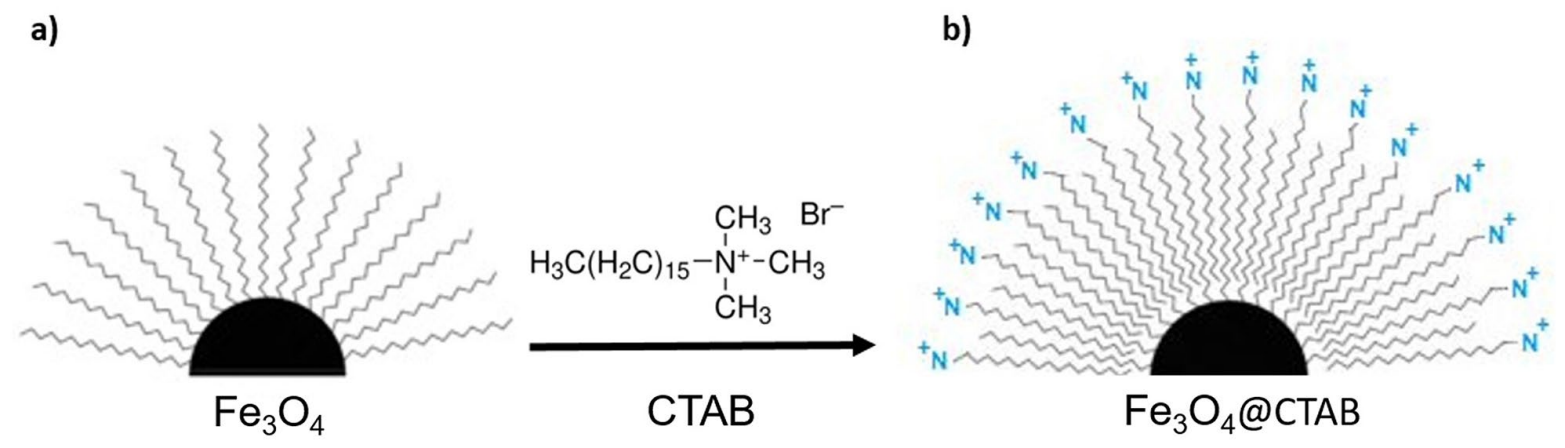
**(a)** Hydrophobic Fe_3_O_4_ NPs coated with oleic acid, and **(b)** hydrophilic Fe_3_O_4_ NPs coated with oleic acid and a layer of CTAB

In order to make the as-synthesized hydrophobic NPs (Fe_3_O_4__A and Fe_3_O_4__B) soluble in water (Fig. 1a) a coating approach based on previously refined protocol was carried out [33]. In this case, instead of using the poly(maleic anhydride-alt-1-octadecene) (PMAO) polymer for the coating the amphiphilic CTAB (Cetyl Trimethyl Ammonium Bromide) molecule was used (Fig. 1b). A CTAB solution in chloroform was added to a 1 mg/mL stock solution of NPs (maintaining a ratio of 100 molecules per nm^2^ of Fe_3_O_4_ NP surface). After stirring the mixture for 15 min, the solvent was evaporated under vacuum and the nanoparticles were dispersed in chloroform. This process was repeated three times, and the last redispersion was carried out using distilled H_2_O. Finally, the two samples functionalized with CTAB (Fe_3_O_4__A@CTAB and Fe_3_O_4__B@CTAB NPs) were further washed with distilled water (3 times) by centrifugation to remove the excess of CTAB that was not attached to the surface of the NPS. The scheme of the NPs coating process has been displayed in Fig. 1.

### Computational methods

**Fig. 2:**
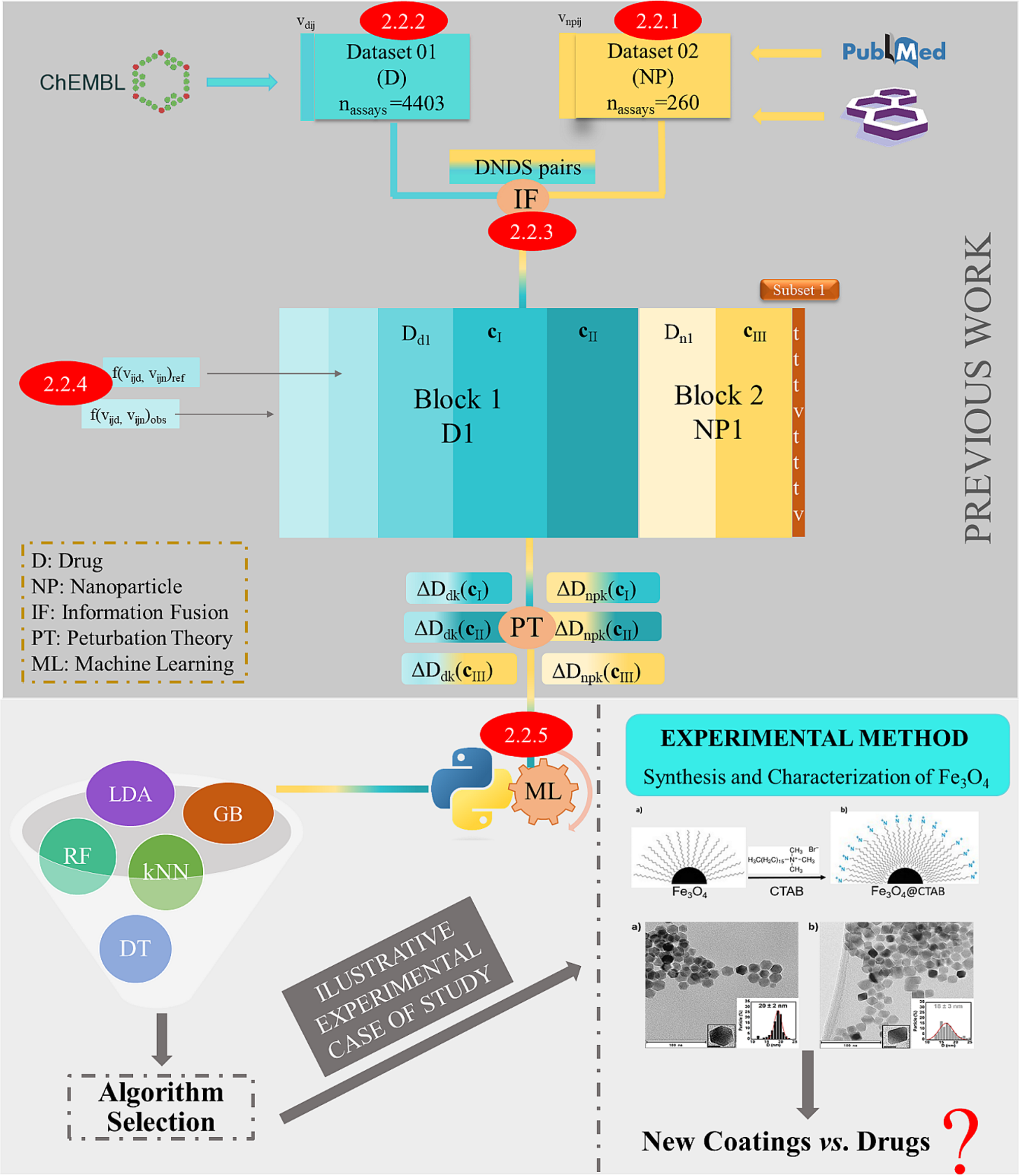
IFPTML detailed information-processing workflow. Step 2.2.1 and 2.2.2 Data collection. Step 2.2.3 Data pre-processing and Information Fusion (NP and NDDs assay). Step 2.2.4 Objective and reference functions definition. Step 2.2.5 PTO calculation

In a previous work, we collected three datasets from different databases. The first dataset (Dataset 01) from ChEMBL, with information from preclinical trials of different NDDs, was merged with Dataset 02 built from NP data collected from the literature. As a result, three large subsets (Subset 1, Subset 2, Subset 3) with different variables were obtained, from which the best IFPTML model for the effective N2D3 systems was obtained [34]. In this work we reprocessed all the information with Python algorithms in order to obtain open access code for this problem for the same time. To construct the IFPTML models, we followed the sequential steps outlined in Fig. 2, which illustrates the overall workflow of the computational procedures employed in this study. Additionally, to facilitate comprehension, each step was annotated with a corresponding enumeration (e.g., 2.2.1, 2.2.2).

#### NP cytotoxicity dataset

Simultaneously, the dataset of preclinical assays for cytotoxicity/ecotoxicity of NPs were collected from 62 papers. (step 2.2.2 in Fig. 2). This dataset contained 260 preclinical assays for 31 NPs, resulting in an average of approximately 8.39 assays per NP. Furthermore, the dataset covered a wide range of NP properties, including morphology, physicochemical properties, coating agents, assay duration, and measurement conditions. These properties were represented as discrete variables (**c**_nj_) used to characterize the conditions and labels of each assay. We categorized all specific conditions of each assay into a general vector **c**_nj_ = [c_n1_, c_n2_, c_n3_ ….c_nmax_]. These variables were biological activity parameters (c_n0_), cell lines utilized in assays (c_n1_), NP shapes (c_n2_), measurement conditions (c_n3_), and coating agents (c_n4_). Please see more details about the dataset content in the Supporting Information SI00.docx, 1.1.1. NP cytotoxicity dataset.

#### NDDs dataset from ChEMBL

At first, 4403 preclinical assays of Neurodegenerative Disease Drugs (NDDs) were downloaded from the ChEMBL database (step 2.2.1. in Fig. 2) [35–37]. The dataset comprised 2566 different NDDs, with an average of around 1.71 assays per drug. Additionally, we defined as categorical variables (c_dj_) the conditions which covered biological activity parameters (c_d0_), target proteins associated with NDDs (c_d1_), cell lines used in NDDs assays (c_d2_), and organisms involved (c_d3_). The nature and quality of the data were also defined as categorical variable, including type of target (c_d4_), type of assay (c_d5_), data curation (c_d6_), confidence score (c_d7_), and target mapping (c_d8_). Additionally, the database provided molecular descriptors (**D**_dk_ = [D_d1_, D_d2_]) to characterize the chemical structure of NDDs compounds. Specifically, two types of molecular descriptors were used for each compound: the logarithm of the n-Octanol/Water Partition coefficient (LOGP_i_) and the Topological Polar Surface Area (PSA_i_). Please see more details about the dataset content in the Supporting Information SI00.docx, 1.1.2. NDDs dataset from ChEMBL.

#### IF process drug nanoparticle delivery system (DNDS) pair resampling

Initially, we utilized the objective value v_ij_ to formulate the IFPTML model. The IFPTML model involved two types of observed values, denoted as v_ij_(c_d0_) and v_nj_(c_n0_), corresponding to both NDDs and NPs. Additionally, we established the target function by employing the descriptor vectors denoted as **D**_dk_ (for the drugs) and **D**_nk_ (for NPs) as input variables in the AI/ML model. In order to simulate a real experiment with the N2D3 systems system, we prioritize certain properties while reducing others. To do this, we defined the desirability value as d(c_d0_) = 1 or d(c_n0_) = 1. This value d(c_d0_) = 1 when we needed to maximize the value of v_ij_(c_d0_) or v_nj_(c_n0_), otherwise d(c_d0_) = -1 or d(c_n0_) = -1. On the other hand, we used the cutoff to rescale the parameters of v_ij_(c_d0_) and v_nj_(c_n0_) to achieve the observed functions f(v_ij_(c_d0_))_obs_ and f(v_nj_(c_n0_))_obs_. These values were obtained as: f(v_ij_(c_d0_))_obs_ = 1, if v_ij_(c_d0_) > cutoff and d(c_d0_) = 1 or v_ij_(c_d0_) < cutoff and desirability d(c_d0_) = -1, f(v_ij_(c_d0_)) = 0 otherwise. Please see more details in the Supporting Information SI00.docx, 1.1.3. IF process DNDS pair resampling.

#### Definition of objectives and reference functions

**Fig. 3:**
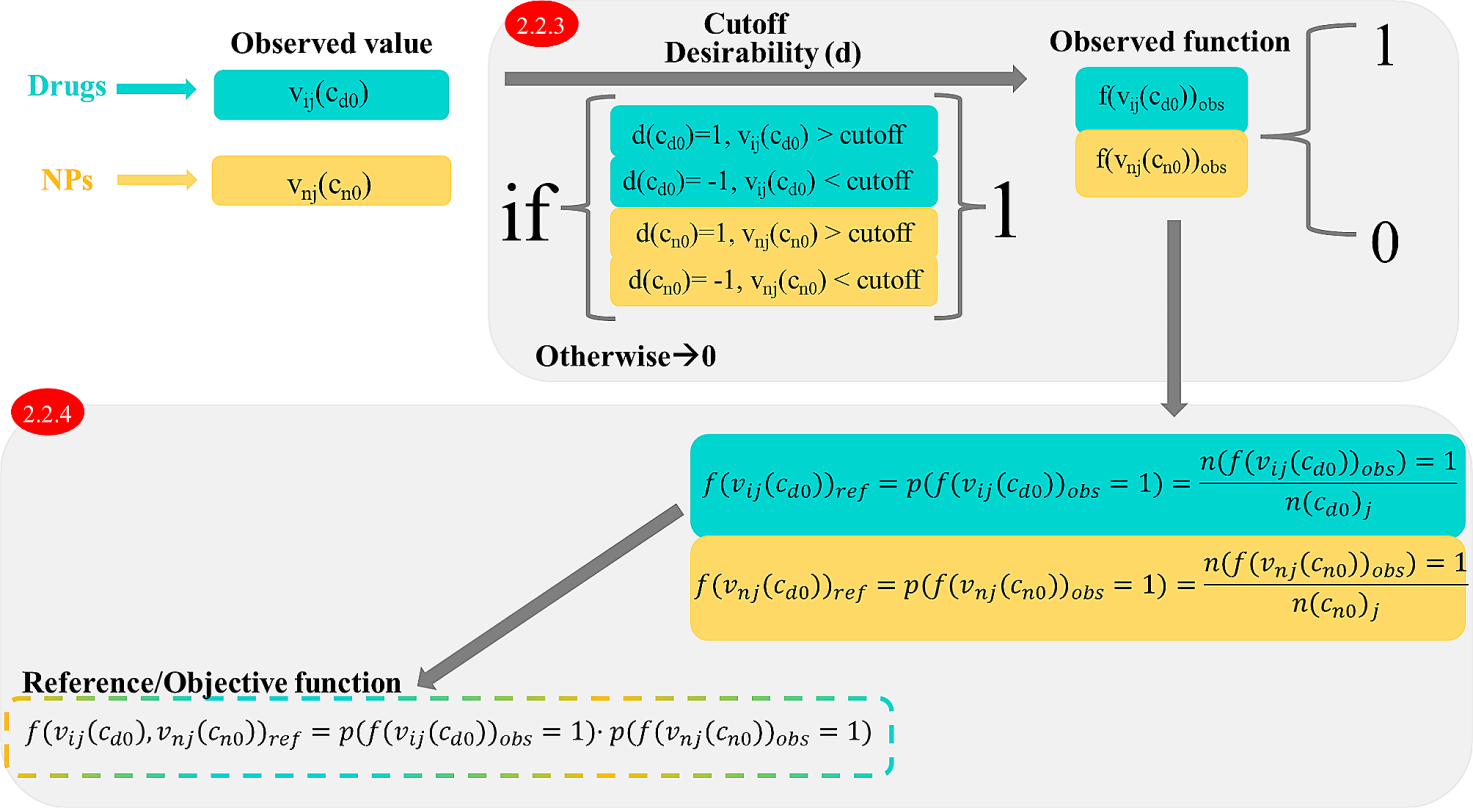
Workflow for the observed and reference function definition (see details in supporting information)

Another input variables of the IFPTML model is the reference/objective function, defined as f(v_ij_(c_d0_), v_nj_(c_n0_))_ref_. The f(v_ij_(c_d0_), v_nj_(c_n0_))_ref_ function defines the expected probability f(v_ij_(c_d0_), v_nj_(c_n0_))_ref_ = p(f(v_ij_(c_d0_), v_nj_(c_n0_))_ref_ = 1) of getting the desired activity for a particular property obtained. The reference function f(v_ij_(c_d0_), v_nj_(c_n0_))_ref_, is calculated as the number of positive outcome n(f(v_ij_(c_d0_)) = 1) (for drugs) and n(f(v_nj_(c_n0_)) = 1) (for NPs) divided by the total number of cases for the NDDs and NP systems individually. These functions are characterized as: f(v_ij_(c_d0_))_ref_ = p(f(v_ij_(c_d0_))_ref_ = 1) = n(f(v_ij_(c_d0_))_ref_ = 1)/n(c_n0_)_j_ and f(v_nj_(c_n0_))_ref_ = p(f(v_nj_(c_n0_))_ref_ = 1) = n(f(v_nj_(c_n0_))_ref_ = 1)/n(c_n0_)_j_. Please see more details in the Supporting Information SI00.docx, 1.1.4. Definition of objectives and reference functions Fig. 3.

#### PTO calculation

##### IFPTML N2D3 systems data analysis phases

The dataset in study was formed by structural descriptors vectors denoted as **D**_nk_ and **D**_dk_, for each NPs [38–40] and NDDs [35, 41–43]. Furthermore, we defined assay condition vectors as **c**_nj_ and **c**_dj_ to denote each label for both NPs and NDDs. For more detail information about the structural descriptors and assay condition vectors, refer to the Supporting Information SI00.docx, 1.1.5. PTO calculation (IFPTML N2D3 systems data analysis phases).

##### Preprocessing of PT data

The IFPTML study incorporates all vectors c_dj_ and c_nj_, representing the non-numerical experimental conditions and labels for both NDDs and NP preclinical assays. Subsequently, we calculated the Perturbation Theory Operators (PTOs), taking into account the Moving Average (MA) of NDDs and NP (see, Eq. 1 and Eq. 2). The PT initiates with the experimental/observed value of an already known activity and adds the perturbations/variations to the system [26, 27, 44–47]. For more detail information, refer to the Supporting Information SI00.docx, 1.1.5. PTO calculation (Preprocessing of PT data).

1 $$\Delta {\rm{D}}\left( {{{\rm{D}}_{{\rm{dk}}}}} \right) = {{\rm{D}}_{{\rm{dk}}}} - \left\langle {{{\left( {{{\rm{D}}_{{\rm{dk}}}}} \right)}_{{{\bf{c}}_{{\rm{dj}}}}}}} \right\rangle$$

2 $$\Delta {\rm{D}}\left( {{{\rm{D}}_{{\rm{nk}}}}} \right) = {{\rm{D}}_{{\rm{nk}}}} - \left\langle {{{\left( {{{\rm{D}}_{{\rm{nk}}}}} \right)}_{{{\bf{c}}_{{\rm{nj}}}}}}} \right\rangle$$

##### NANO.PTML models training and validation overview

In developing the model using ML techniques, each sample case is categorized into either the training (subset = t) or validation (subset = v) series. The assignment process of cases should be random, representative, and stratified [48, 49]. Subsequently, we divided the cases into three equal parts for subset = t (training) and one-quarter for subset = v (validation) for the whole dataset. It is important to note that the 75% and 25% proportion kept between training and validation [48]. Additionally, the performance of the NANO-PTML models was evaluated using different statistical metrics, particularly Sensitivity (Sn) and Specificity (Sp) [50, 51]. For more detail information, refer to the Supporting Information SI00.docx, 1.1.5. PTO calculation (NANO.PTML models training and validation overview).

##### NANO.PTML simulation of experimental case of study

We conducted a computational analysis to illustrate the applicability of the NANO.PTML model in an example of a real wet-laboratory setting. In this context, we predicted the Fe_3_O_4_-core based NPs with CTAB as the coating system, as reported in the experimental part here. To create a more ambitious prediction experiment, we added multiple combinations of Fe-based cores, coatings, cell lines, and shapes. Particularly, this prediction dataset was formed by diverse combinations of up to 123 drugs, 53 cell lines, 16 coats, 5 NPs core and 5 NP shapes. The NPs core studied were CoFe_2_O_4_, ZnFe_2_O_4_, Fe_3_O_4_, Fe_2_O_3_ and Fe. Additionally, the cell lines used in the cytotoxicity predictive study were L929 (M), HepG2 (H), A549 (H), among other. On the other hand, the organisms used in the eco-toxicity computational study were *Vibrio fischeri*, *Oryzias latipes (embryos)*, etc. Furthermore, there were different NP shapes such as irregular, elliptical, etc. Finally, the NP coating agents studied in this research were Polyvinyl alcohol (PVA), Polyvinylpyrrolidone (PVP), CTAB, potato starch (PS), PEG-Si(OMe)_3_ (PEG), etc. For more detail information of simulation experiment, refer to the Supporting Information SI00.docx, 1.1.5. PTO calculation (NANO.PTML simulation of experimental case of study).

## Results and discussion

### AI/ML python computational models

**Fig. 4:**
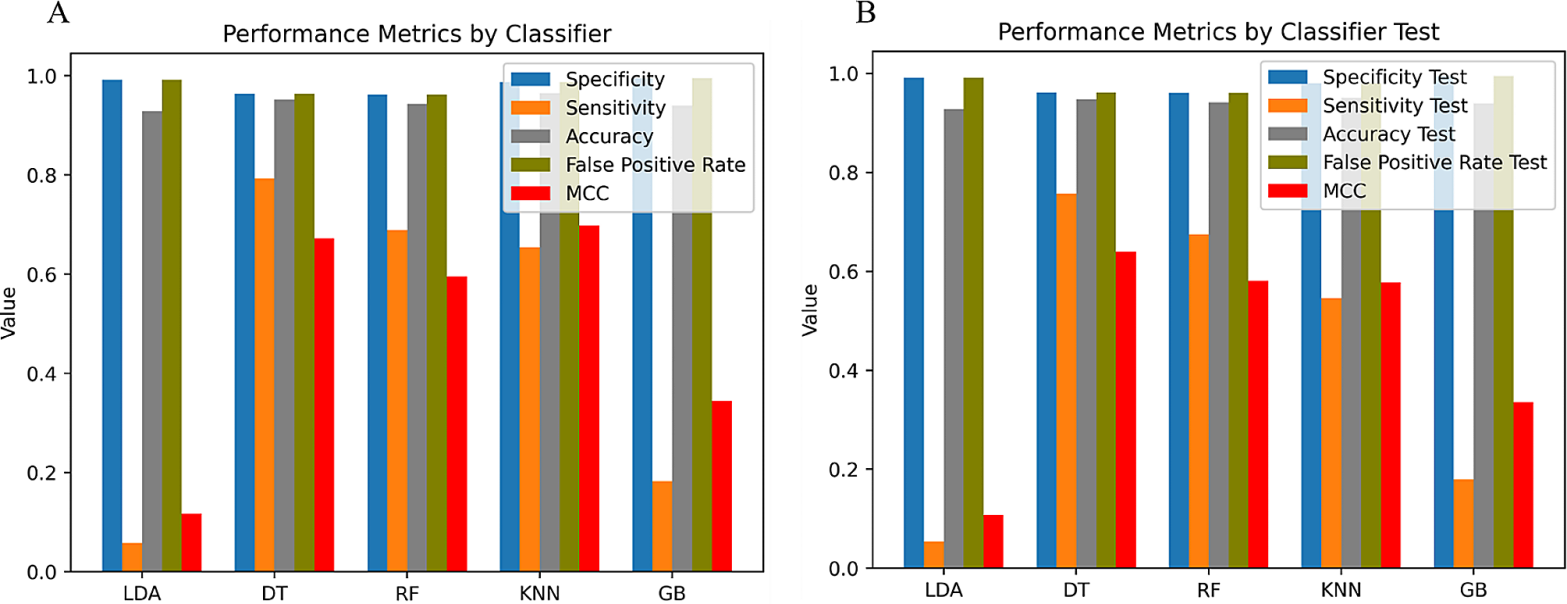
Summary of the statistical parameters obtained for the linear and non-linear NANO.PTML models. (**A**) Training set and (**B**) Validation set

In order to design AI/ML models for predicting the NP system as a neurodegenerative drug carrier, the Scikit-Learn module in Python [52] was used to identify the best AI/ML estimator. In this context, linear and non-linear classifiers were employed, specifically, Linear Discriminant Analysis (LDA) [53], Decision Tree (DT) [54], Random Forest (RF) [55], k-Nearest Neighbor (kNN) [56], and Gradient Boosting (GB) [57]. Additionally, the Expert-Guided Selection (EGS) [34] approach was employed to identify the most significant variables capable of defining the NANO.PTML system. The variables utilized for these models were considered crucial for describing the NANO.PTML system: ΔDPSA(**c**_I_)_dj_ (deviation of topological Polar Surface Area) for neurodegenerative drug and for NPs as drug carrier the variables including ΔDt(**c**_III_)_nj_ (deviation of NP safety time), ΔDLnp(**c**_III_)_nj_ (deviation of NP length), ΔDVnpu(**c**_III_)_nj_ (deviation of NP core volume), ΔDVxcoat(**c**_III_)_nj_ (deviation of McGowan volume), and ΔDVvdwMGcoat(**c**_III_)_nj_ (deviation of van der Waals volume from McGowan volume) were taken into account. Table 1; Fig. 4 presented the statistical parameters obtained by linear and non-linear models. The results showed that the DT classifier exhibited a good fit in both the training and validation sets, with Specificity (Sp) values of 96.4/96.2 and Sensitivity (Sn) values of 79.3/75.7, respectively. Another important statistical parameter included is the Mathew’s Correlation Coefficient (MCC) values [58], giving 0.6722/0.6401 in training/validation series.

**Table 1:** Statistical parameters used for NANO.PTML models

Data			Stat. Param.	Param.		Sub Set Predicted	
Algorithm	Set	Subset	MCC	(%)		0	1
LDA	t	0	0.1171	Sp	99.2	346,697	2841
		1		Sn	5.8	23,992	1470
	v	0	0.1076	Sp	99.2	115,555	981
		1		Sn	5.4	8006	458
DT	t	0	0.6722	Sp	96.4	336,788	12,750
		1		Sn	79.3	5277	20,185
	v	0	0.6401	Sp	96.2	112,058	4478
		1		Sn	75.7	2060	6404
kNN	t	0	0.6986	Sp	98.7	344,997	4541
		1		Sn	65.4	8801	16,661
	v	0	0.5779	Sp	98.0	114,243	2293
		1		Sn	54.5	3849	4615
RF	t	0	0.5952	Sp	96.3	336,418	13,120
		1		Sn	68.4	8057	17,405
	v	0	0.5813	Sp	96.2	112,057	4479
		1		Sn	67.0	2794	5670
GB	t	0	0.3443	Sp	99.5	347,761	1777
		1		Sn	18.3	20,808	4654
	v	0	0.3355	Sp	99.5	115,898	638
		1		Sn	17.9	6946	1518

After tuning the hyperparameters to develop the DT algorithm which play a crucial role in determining its performance and behavior [59]. The best combination found were the following; The ccp-alpha parameter, set to 0.0, controls the complexity of the tree by correcting excessive branching and preventing overfitting. The class-weight parameter assigns weights to different classes within the dataset, in this case we set class 0 at 40% and class 1 at 60%, addressing potential imbalances in class distribution. The choice of criterion as “gini” indicates the use of Gini impurity as the measure of split quality, influencing how the tree partitions the feature space. Furthermore, max-depth is set to 15, limiting the depth of the tree to prevent it from growing overly complex and overfitting to the training data. The max-features and max-leaf-nodes parameters, both set to “None”, which allow the tree to explore all available features and leaf node possibilities, respectively, without imposing additional constraints. The min-impurity-decrease set at 0.0 defines the minimum impurity decrease required for a split, regulating the tree’s growth. The min-samples-leaf and min-samples-split, both set to 5 and 2 respectively. These parameters establish the *minimum* number of samples required in a leaf node or for a node split, contributing to the ability of generalizing the tree and avoiding it from being overly specific to the training data. The min-weight-fraction leaf was set to 0.0, indicating that it was not applied, while the random-state was set to 42, ensuring reproducibility of results across different runs of the model. Finally, splitter as “best” indicates that the best split at each node is determined based on the chosen criterion, enabling optimal tree construction. Further information about these parameters can be found in the documentation provided by the Scikit-learn library [52]. The hyperparameter used for LDA, kNN, etc. can be found in Table S1 Supporting Information SI00.docx.

**Fig. 5:**
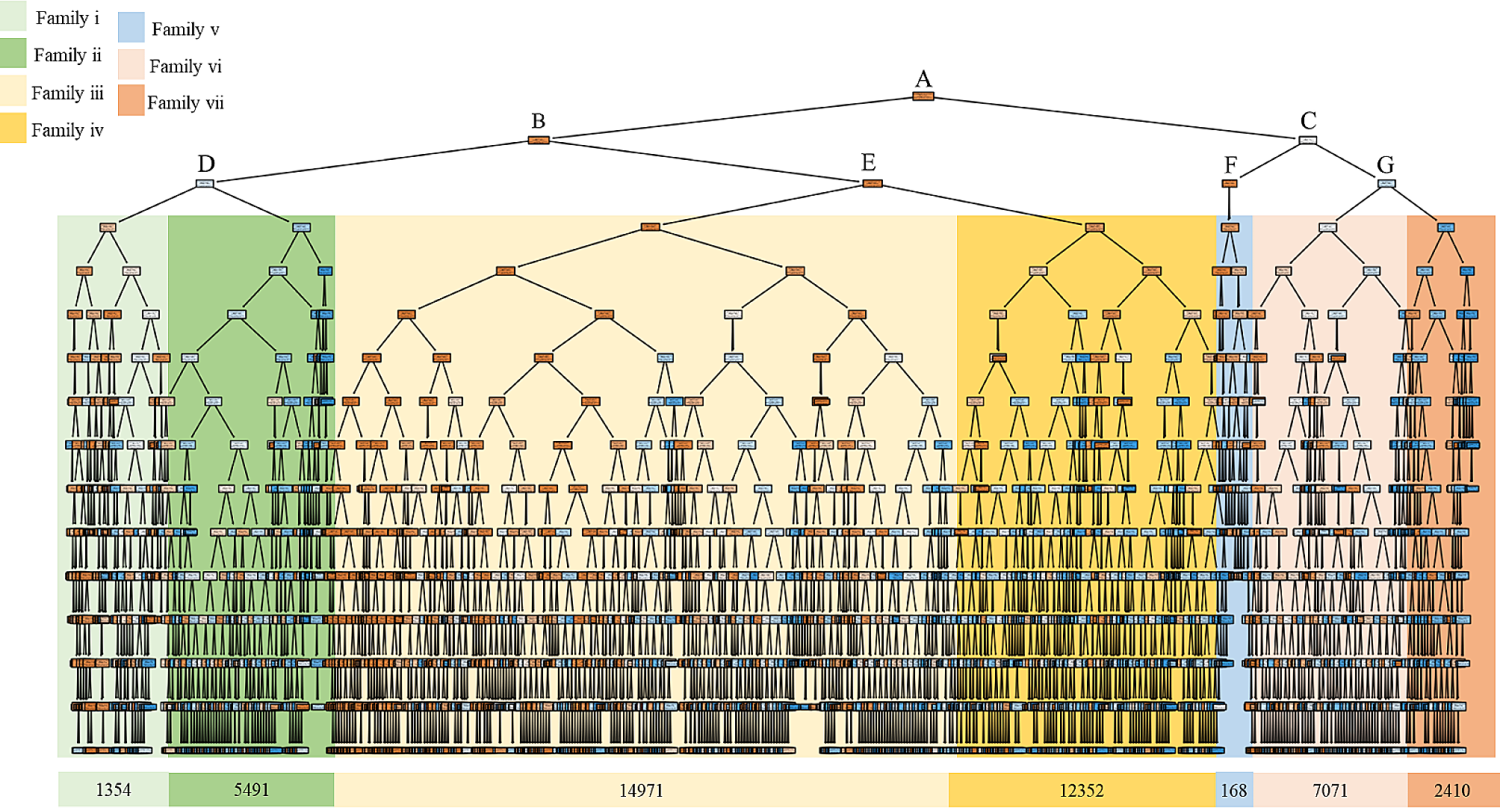
Plot for DT structure

Figure 5 depicts the structure of the decision tree, comprising 3249 nodes with a depth of 15 layers and terminating in 1625 leaf nodes. Final predictions or decisions are made based on the input data [60]. To facilitate better understanding of this tree plot, we have focused the explanation on a tree depth of 2 layers, resulting in 4 leaf nodes, which collectively form 7 main families. This analysis involved input variables such as ΔDVvdwMGcoat(**c**_III_)_nj_, f(v_ij_(c_d0_),v_nj_ (c_n0_))_ref_, and ΔDLnp(**c**_III_)_nj_. Full information of the description for each family can be seen in Table 2. For example, in family i, composed by NPs with lower McGowan volume deviation than Families v-vii and lower prior probability of activity than families ii-iv.

**Table 2:** Description of the 7 main families within the DT structure. The color of each family consistently matches that depicted in Fig. 4

Depth			Family Description	
0	1	2	Left	Right
ΔDVvdw MGcoat(**c**_III_)_nj_ ≤ 115.27 **A**	ΔDVvdw MGcoat(**c**_III_)_nj_ ≤ -122.477 (**Left**) **B**	f(v_ij_(c_d0_), v_nj_ (c_n0_))_ref_ ≤ 0.078 (**Left**) **D**	**Family i, composed by NPs with lower McGowan volume deviation than Families v-vii and lower prior probability of activity than families ii-iv.** Overall, this implies smaller NPs, possibly with lower polarizability, and lower expected biological property values suggesting overall reduced drug-NP activity likelihood. The 0.4% (1354/375,000) of cases are predicted as class 1. Consequently, NPs in this family should not be short-list for assay according to the model.	**Family ii, composed by NPs with higher McGowan volume deviation than Family i and lower prior probability of activity than families iii, iv.** Overall, this implies larger NPs, possibly with higher polarizability, and lower expected biological property values for Drug and NP suggesting overall increased activity likelihood. The 1.5% (5491/375,000) of cases are predicted as class 1. Consequently, NPs in this family should not be short-list for assay according to the model.
		f(v_ij_(c_d0_), v_nj_(c_n0_))_ref_ ≤ 0.118 (**Right**) **E**	**Family iii, composed by NPs with lower McGowan volume deviation than Families v-vii and lower prior probability of activity than family iv.** Overall, this implies smaller NPs, possibly with lower polarizability, and low to medium expected biological property values suggesting overall reduced drug-NP activity likelihood. The 4% (14,971/375,000) of cases are predicted as class 1. Consequently, NPs in this family should be short-list for assay according to the model.	**Family iv, composed by NPs with higher McGowan volume deviation than family iii and higher prior probability of activity than family i-iii.** Overall, this implies larger NPs with higher polarizability. Medium to high biological property values indicate a higher likelihood of drug-NP activity. The 3.3% (12,352/375,000) of cases are predicted as class 1. Consequently, NPs in this family should be short-list for assay according to the model.
	f(v_ij_(c_d0_), v_nj_(c_n0_))_ref_ ≤ 0.057 (**Right**) **C**	ΔDt(**c**_III_)_nj_ ≤ -8.0 (**Left**) **F**	-	**Family v, composed by NPs with higher McGowan volume deviation and prior probability of activity than family i-iv.** Overall, this implies larger NPs with low to medium biological property values show increases drug-NP activity likelihood and the deviation of synthesis of NPs time shorter than the average NPs in the dataset. The 0% (168/375,000) of cases are predicted as class 1. Consequently, NPs in this family should not be short-list for assay according to the model.
		f(v_ij_(c_d0_), v_nj_(c_n0_))_ref_ ≤ 0.103 (**Right**) **G**	**Family vi, composed by NPs with higher McGowan volume deviation i-v and lower prior probability of activity than family vii.** Overall, this implies larger NPs with low biological property values show reduced drug-NP activity likelihood. The 1.9% (7071/375,000) of cases are predicted as class 1. Consequently, NPs in this family should be short-list for assay according to the model.	**Family vii, composed by NPs with higher McGowan volume deviation and prior probability of activity than families vi.** Overall, this implies larger NPs with low to medium biological property values show increases drug-NP activity likelihood. The 0.6% (2410/375,000) of cases are predicted as class 1. Consequently, NPs in this family should be short-list for assay according to the model.

Overall, this implies smaller NPs, possibly with lower polarizability, and lower expected biological property values suggesting overall reduced drug-NP activity likelihood. The 0.4% of cases are predicted as class 1. Consequently, NPs in this family should not be short-list for assay according to the DT model. However, on the right section of the DT, family ii, composed by NPs with higher McGowan volume deviation than Family i and lower prior probability of activity than families iii and iv. General, this indicates larger NPs, possibly with higher polarizability, and lower expected biological property values for Drug and NP suggesting overall increased activity likelihood. The 1.5% of cases are predicted as class 1. Therefore, NPs in this family should not be short-list for assay according to the model. However, families iii and iv yielded more promising results, with 4% and 3.3% of class 1, respectively. Family iii suggests smaller NPs, possibly with lower polarizability and low to medium expected biological property values, indicating an overall reduced likelihood of drug-NP activity. Conversely, family iv suggests larger NPs with higher polarizability. Medium to high biological property values indicate a higher likelihood of drug-NP activity.

**Fig. 6:**
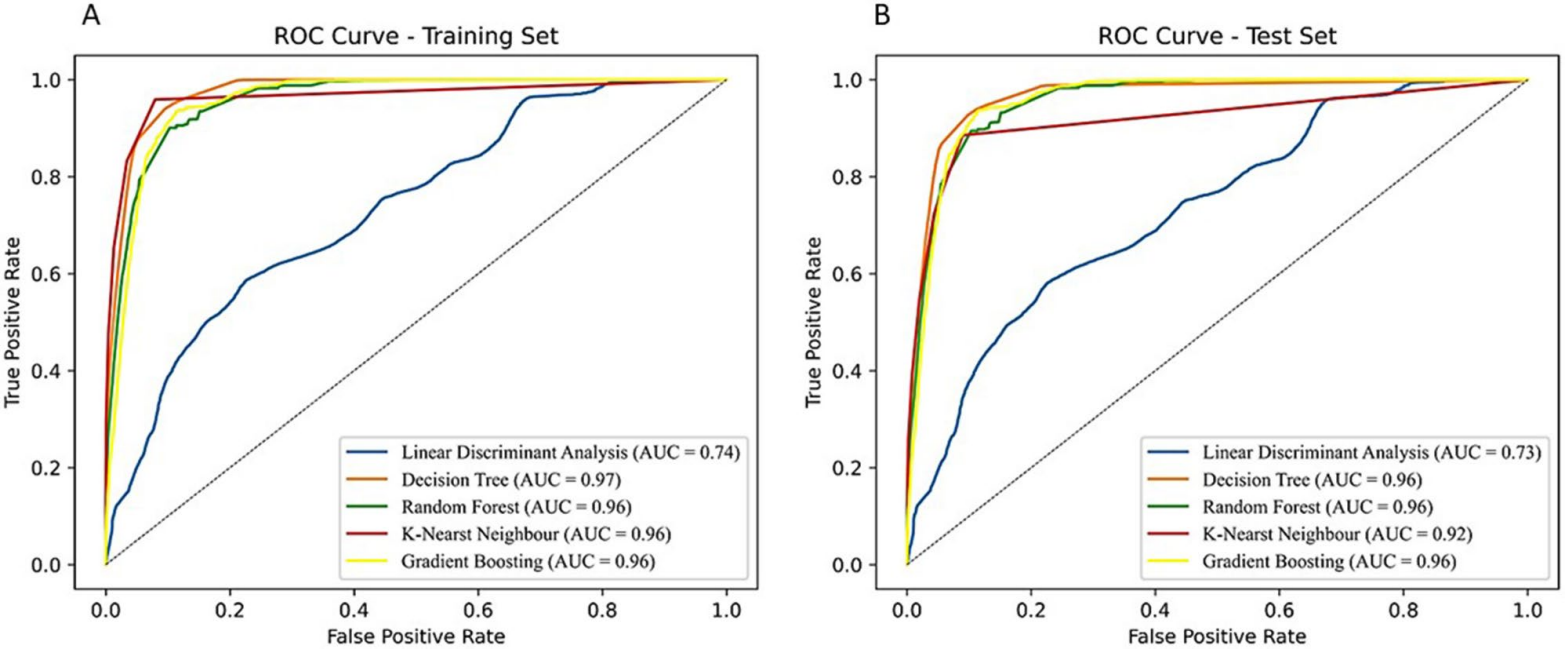
AUROC exploration of NANO.PTML models in both training (A) and validation (B) set

Another statistical metric used in this study is the Area Under Receiver Operating Characteristic (AUROC), for both training and validation set, see Fig. 6 [48]. A high AUROC value indicates better overall performance of the model in terms of its ability to correctly classify instances from both classes. An AUROC of 1.0 represents a perfect classifier, while an AUROC of 0.5 indicates a classifier that performs no better than random guessing [48]. The highest AUROC values, 0.97 − 0.96, are obtained by the DT algorithm, which accordingly matches the results of Sn/Sp in the training/validation set. Whereas, the LDA algorithm is not among the top-performing classifiers, with AUROC values ranging from 0.73 to 0.74.

#### Contrast with earlier AI/ML algorithms

Other research jobs have showed in the recent investigation a wide variety of problems relating with NPs and/or NDDs discovery, see Table 3. Actually, the majority of these researches explore the cytotoxicity of NP assays or NDDs against a large number of species by applying NANO.PTML models. Nevertheless, to the best of our knowledge, there are not study that includes both NP and NDDs component simultaneously or the opportunity of developing N2D3 systems. For example, Kleandrova et al. developed an combined QSTR-perturbation model to simultaneously explore ecotoxicity and cytotoxicity of NPs under different experimental conditions, including diverse measures of toxicities, multiple biological targets, compositions, sizes and conditions to measure those sizes, shapes, times during which the biological targets were exposed to NPs, and coating agents [44]. The model was obtained from 36,488 cases of NP-NP pairs. Nevertheless, in this research Kelandrova et al. is only restricted to the study of ecotoxicity and cytotoxicity of NPs and does not contemplate the data about NDDs components. Similarly, Cordeiro et al. built up the QSAR-perturbation model which involves 5520 cases (NP–NP pairs). The aim of this model is the simultaneous prediction of the ecotoxicity of NPs against several assay organisms (bio-indicators), by considering also multiple measures of ecotoxicity, as well as the chemical compositions, sizes, conditions under which the sizes were measured, shapes, and the time during which the diverse assay organisms were exposed to nanoparticles [40]. As the previous model, they do not take into account the NDDs biological activity. On the other hand, Luan et al. generated the mx-QSAR model from 4915 cases of multiple assays of neurotoxicity/neuroprotective effects of drugs. In addition, the model was trained with a dataset which involved diverse assay endpoints of 2217 compounds. Each compound was assayed in at least one out of 338 assays, which included 148 molecular or cellular targets and 35 standard type measures in 11 model organisms (including human).Unlike previous models, this mx-QSAR algorithm contained information NDDs, however, it does not consider the NP as part of this system [61]. In this paper, we developed an innovative system including both NP and NDDs components. The results of the NANO.PTML-DT was quite satisfactory Sp values of 96.4/96.2 and Sn values of 79.3/75.7 in training and validation series including 375 K and 125 K cases, respectively. Other research with similar scope as the present work, García et al. built up the LDA linear model in order to predict the results of 42 different experimental tests for GSK-3 inhibitors with heterogeneous structural patterns. GSK-3β inhibitors are interesting candidates for developing anti-Alzheimer compounds among others urgent diseases. These authors obtained Sn/Sp ≈ 90% in training/validation series [62]. On the other hand, Ferreira da Costa et al. constructed LDA model so as to predict the properties of a query compound or molecular system in experimental assays with multiple boundary conditions involved in the dopamine pathway. They obtained Sn/Sp ≈ 70–91% in both training and validation series [63]. However, it is worth mentioning that the contract of statistical parameters between the model of this work and the previous one is not informative at all due to the fact that the design of each model is specific to the problem to be dealt with.

**Table 3:** NDDs and NP cytotoxicity study using AI/ML algorisms in previous research works

**m**	**Prop.** ^**a**^	**NP**	***n*** ^**b**^	**Var**.^**c**^	**MO** ^**d**^	**AI/ML** ^**e**^	**NP** ^**e**^	**NDDs** ^**e**^	***N*** + **D**^**e**^	**Ref**.^**f**^
1 1	Cytotoxic.	Silica, Nickel and Nickeloxide	1261	5	yes	LDA	yes	no	no	[44]
2	Cytotoxic.	Nickel	4133	7	yes	LDA	yes	no	no	[40]
3	Cytotoxic.	Metal oxide	17	1	no	MLR	yes	no	no	[64]
4	Cytotoxic.	Fullerene derivatives	52	5	yes	MLR	yes	no	no	[65]
5	Cytotoxic.	TiO_2_	26	1	yes	MLR	yes	no	no	[66]
**m**	**Prop**.^**a**^	**NDDs**	**n** ^**b**^	**Var**.^**c**^	**MO** ^**d**^	**AI/ML** ^**e**^	**NP** ^**e**^	**NDDs** ^**e**^	**N + D** ^**e**^	**Ref.** ^**f**^
6	Neuro-protec	Peptidomimetics	41,082	9	yes	LDA	no	yes	no	[63]
7	Neuro-protec.	Dipropargylated derivatives	4915	3	yes	LDA	no	yes	no	[61]
8	anti-Alzhe.	GSK-3beta inhibitors	4508	5	yes	LDA	no	yes	no	[62]
9	Neuro-protec.	Heterogeneous	2661	5	yes	LDA	no	yes	no	[67]
10	Neuro-protec.	Heterogeneous	11,051	5	yes	ANN	no	yes	no	[68]

^a^Biological activity properties experimentally measured

^b^Number of cases used for building up the model

^c^Number of variables involved in the AI/ML techniques

^d^Multi-outputmodel

^e^LDA= Linear Discriminant Analysis; SVM = Support Vector Machine; MLR = Multiple Linear Regression; Het. Heterogenerous drug neurodegenerative database. The NDDs, NP or both components included in the resulting model

^f^Reference

### Experimental study of new system

#### Characterization of Fe_3_O_4_nanoparticles

**Fig. 7:**
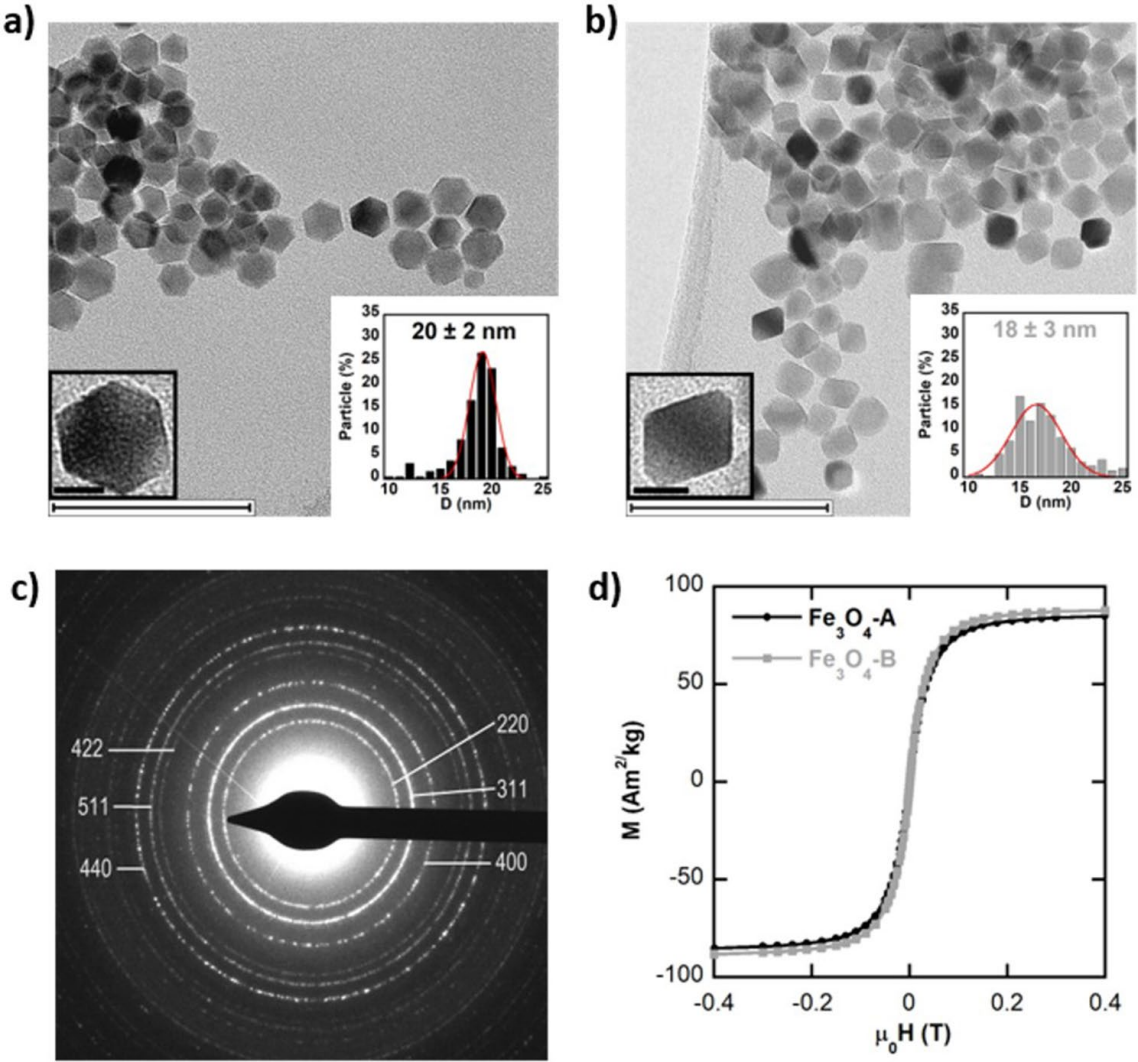
TEM micrographs of **(a)** Fe_3_O_4__A (cuboctahedral) and **(b)** Fe_3_O_4__B (octahedral) nanoparticles. Large scale bars: 100 nm. Zoom scale bars: 10 nm, **(c)** a representative Electron Diffraction (ED) pattern corresponding to sample Fe_3_O_4__A and **(d)** M (H) curves of both samples at room temperature

Initially the hydrophobic NPs (samples Fe_3_O_4__A and Fe_3_O_4__B) have been structurally, morphologically and magnetically characterized (Table 4). Both samples present the inverse spinel structure of magnetite (Fe_3_O_4_, S.G. Fd-3 m) with no traces of secondary phases. The crystallite sizes of the samples were calculated from the maximum diffraction peak (311) of X-ray powder diffraction patterns using Scherrer’s equation. The calculated crystallite sizes of the two samples are around 24 nm and are compatible with the average physical size determined by TEM analysis (see Table 4; Fig. 7). The rather good agreement between the two techniques (DRX and TEM) indicate that the NPs of both samples are composed of single nanocrystals. In relation to the morphology of the NPs, sample Fe_3_O_4__A is composed of NPs with more facets (cuboctahedrons), while the NPs of sample Fe_3_O_4__B present octahedral-like shape as it can be seen in Fig. 7a) and b), respectively.

**Table 4:** Summary of the features of the two Fe_3_O_4_ NP samples: Weight% of the organic matter (O.M.%) in the as-synthesized hydrophobic NPs, size of crystalline domain (D_DXR_) by Scherrer calculation from the main (311) diffraction peak, the average dimension of the inorganic core obtained by TEM (D_TEM_), saturation magnetization (M_S_) of the inorganic core at RT and the hydrodynamic size (D_H_) and Z potential (Z) of the hydrophilic NPs coated with CTAB

Experimental Parameters	Sample	
	Fe_3_O_4__A	Fe_3_O_4__B
O.M. (%)	28	28
D_DRX_ (nm) 311	25 ± 1	24 ± 1
D_TEM_ (nm)	20 ± 2	18 ± 3
Ms (Am^2^/kg)	88	91
Coating	Fe_3_O_4__A@CTAB	Fe_3_O_4__B@CTAB
D_H_ (nm)	72 ± 7	133 ± 38
Z (mV)	5	12

The magnetization dependence with the magnetic field (M(H)) in the two samples has been carried out by DC Magnetometry at RT. The M(H) curves of Fig. 7d display saturation magnetizations (M_S_) of 88 and 91 Am^2^/kg_Fe3O4_, respectively, which proves the high quality of the magnetite phase and the purity of the inorganic core. After coating the hydrophobic NPs with CTAB, both samples (Fe_3_O_4__A@CTAB and Fe_3_O_4__B@CTAB NPs) become highly soluble in water as it is shown by the Z potential values, which are positive due to the cationic nature of the CTAB molecule (see Table 4; Fig. 7b). Regarding the degree of agglomeration of the NPs in water dispersion, it can be claimed that these NPs are arranged in small clusters (2–5 NPs) because they present moderate hydrodynamic diameters (see Table 4) in comparison to the average diameter of a single NP determined by DRX and TEM.

This experimental section is focused specifically on the NP core of Fe_3_O_4_ with two shapes (cuboctahedral and octahedral) and on the CTAB coating. We performed a computational analysis to demonstrate the practical application of the NANO.PTML model using a real-world wet-laboratory scenario. Additionally, we carried out a simulation experiment that try to mimic this experimental part. For this purpose, we created a prediction dataset with various combinations of NP systems including NP cores, coating agents, cell lines, shapes, and anti-neurodegenerative drugs linked with certain coatings. It is important to note that the total number of combinations, considering NP cores, cell lines, shapes, coating agents, and anti-neurodegenerative drugs, amounted to N_tot_ = n(NP cores) · n(cell lines) · n(NP shapes) · n(NP coats) · n(drugs) = 5 · 53 · 5 · 16 · 123 = 2,607,600 assays. Performing all these combinations in a wet-laboratory is impractical, time-consuming, and resource-intensive. Even with expert criteria, the number of assays remains unmanageable for study. Therefore, the NANO.PTML-DT approach is introduced to address this issue by reducing the number of assays and serving as a guide for the experimental part, highlighting the most promising combinations within the NP systems as drug carriers for neurodegenerative diseases.

### Experimental vs. computational illustrative case of study

#### NANO.PTML-DT simulation experiment

**Fig. 8:**
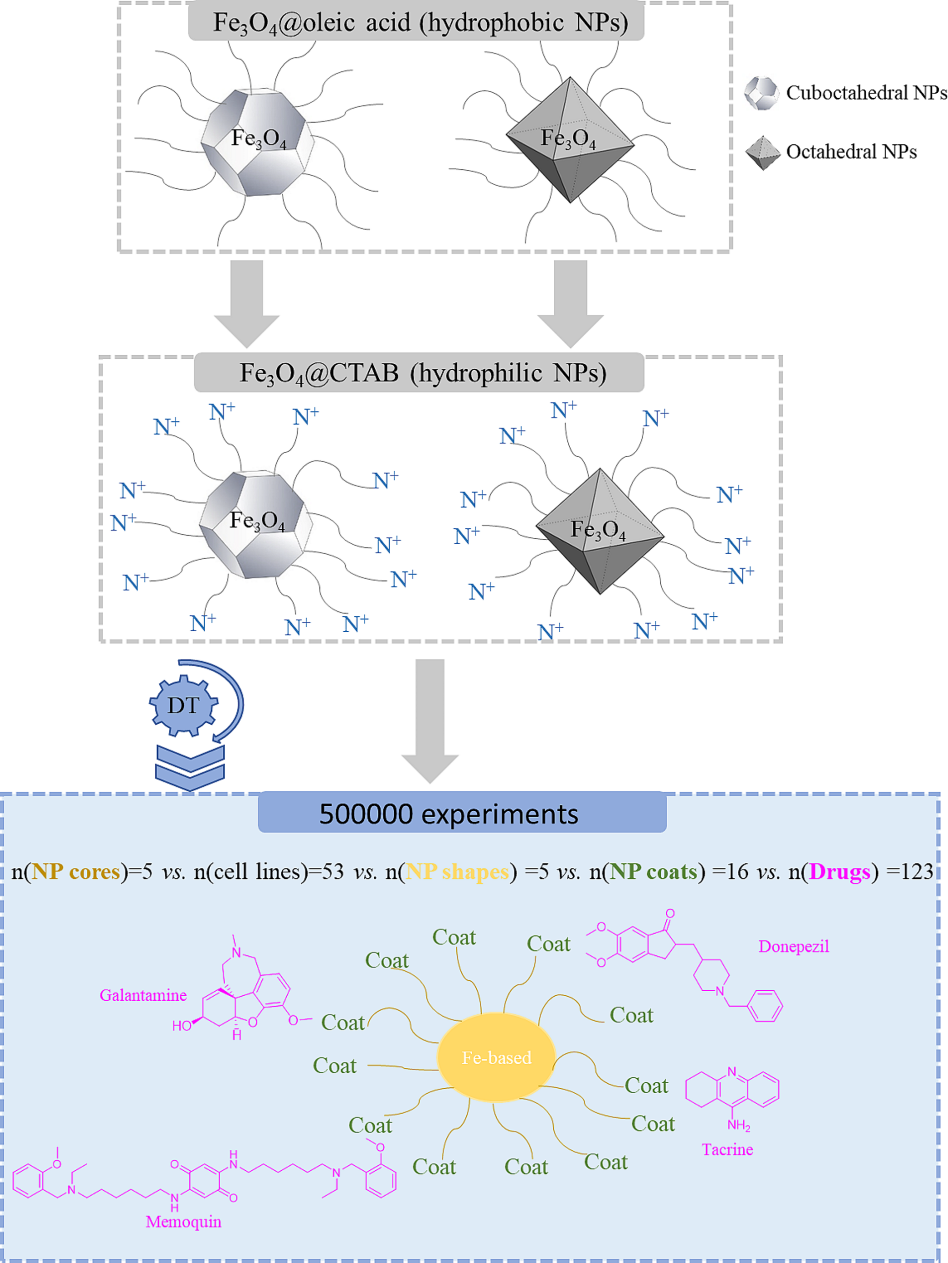
Workflow of experimental illustrative simulation experiment using NANO.PTML approach

In this section, a computational case study was presented to simulate the Fe_3_O_4__A@CTAB and Fe_3_O_4__B@CTAB NPs from the experimental study detailed in this paper (Fig. 8). The aim of this simulation experiment was to forecast the best combination of the NPs core vs. cell lines (cytotoxicity or ecotoxicity) vs. shapes vs. coating agents as mentioned in the previous section. In this scenarios, we created a total of 500,000 assays as new prediction dataset, which was formed by up to n(NPs core) = 5, n(cn1 = cell lines) = 53, n(cn2 = NP shapes) = 5, n(NPs coat) = 16 and n(drugs) = 123.

**Fig. 9:**
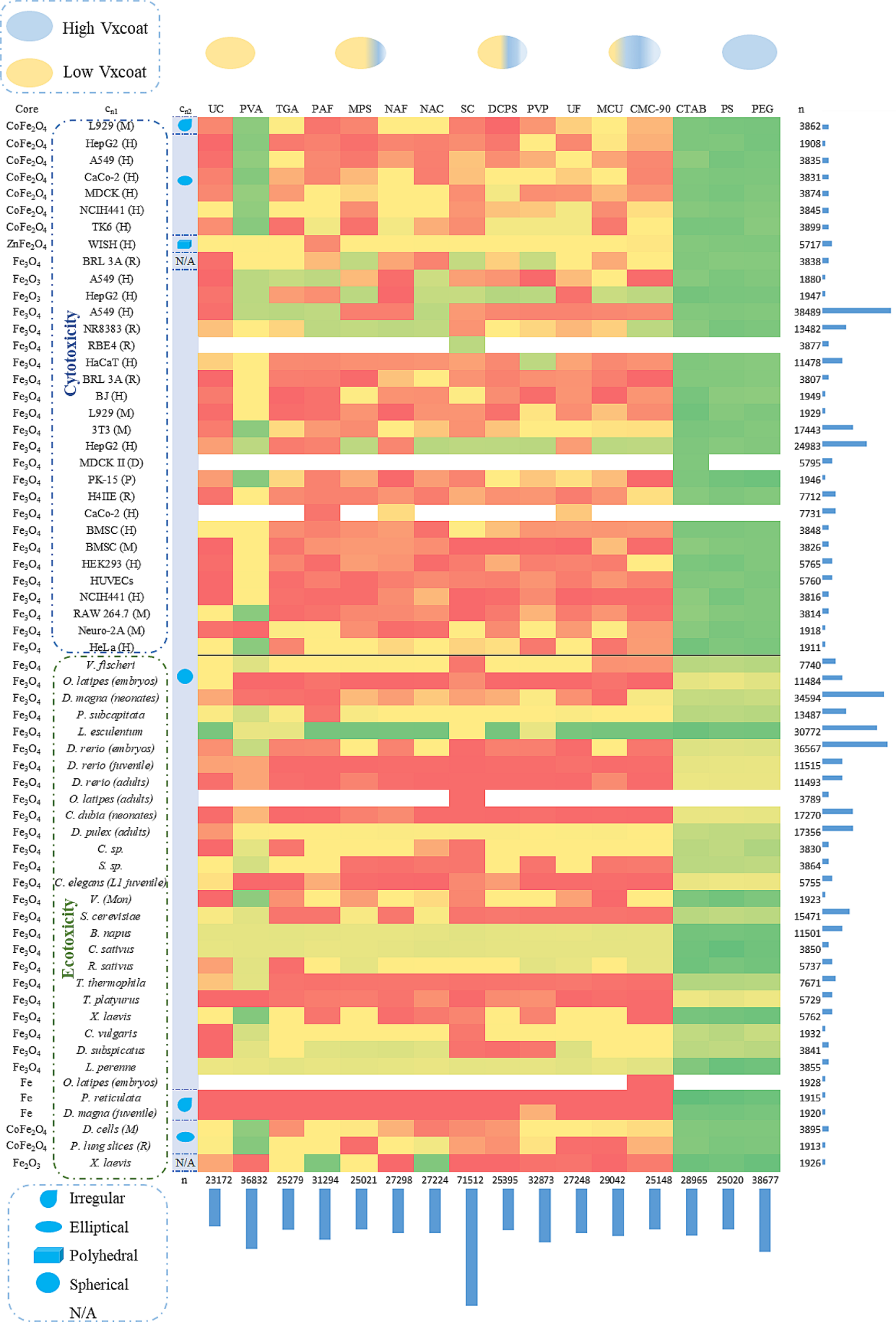
NANO.PTML systems experiment simulation

On the other hand, the DT model was selected due to the good performance of the statistical parameters in both training and validation set, as shown in Table 1. The probability p(NANO.PTML_in_)_**c**nj_ values were acquired with NANO.PTML_in_ system. The heatmap shown in Fig. 9 illustrates the findings using a 3-color scale based on probability values: the green zone represents a high probability range, the yellow zone signifies a moderate to low probability range, and the red zone indicates a very low predicted probability. Assays that had never been reported previously or had very low representation in the original dataset, as well as insignificant combinations of NP systems were depicted in white to prevent overestimation in the results. Additionally, the columns of this heatmap represented the NP core, cell lines, and NP morphology. The column for cell lines was further categorized into cytotoxicity and ecotoxicity. The rows of the heatmap corresponded to the NP coats studied, arranged based on their MacGowan volume_n values. Furthermore, the heatmap contained information regarding the frequency of each combination appearing in both the columns and rows within the prediction dataset.

The prediction was carried out taking into account the cytotoxicity and eco-toxicity. It is crucial the study of the cytotoxicity as NPs are increasingly employed in medical diagnostics and therapies to enhance our comprehension, detection, and treatment of human diseases. The exposure of NPs in consumer products or their use in emerging biomedical applications, such as drug delivery, biosensors, [69] or imaging agents, [70] entails direct ingestion or injection into the body [71]. Additionally, the study of eco-toxicity is critical for assessing their impact on ecosystems, wildlife, and human health [72]. It helps in understanding how NPs interact with the environment, entering food chains and potentially affecting biodiversity.

In this context, the outcomes of the DT model highlighted certain NANO.PTML systems as promising candidates for further investigation. Interestingly, the high prediction value of *Lycopersicon esculentum* proved to be a favorable ecotoxicity cell line, exhibiting high probability values with the majority of coating systems. Contrarily, the least propitious cell lines were *Danio rerio (embryos)*, *Danio rerio (juvenile)*, *Danio rerio (adults)*, *Oryzias latipes (adults), Ceriodaphnia dubia (neonates), Daphnia pulex (adults), Chlorella sp.*, and *Scenedesmus sp.*, which yielding in medium to low probability values. On the other hand, one more important characteristic is MacGowan volume which has been widely used in many areas to estimate the physicochemical and biochemical properties of molecules, [73, 74] specifically for CTAB, PS, and PEG as coating agents, with an exception in PVA. The combination of elliptical-shaped NPs with PVA as a coating agent in cytotoxicity cell lines appears to be a promising candidate for further synthesis. Another important factor is the type of the cell line which obtained higher probability value with cytotoxicity. It is important to note that all predictions generated by this method should be approached with caution and necessitate experimental validation. The NANO.PTML-DT method holds potential for accelerating experimental studies and offering cost-effective preliminary results for a vast database of NANO.PTML systems. This methodology presents an effective and robust tool for guiding experimental research, offering an alternative to laborious trial-and-error testing.

#### General applications of NANO.PTML-DT model

**Fig. 10:**
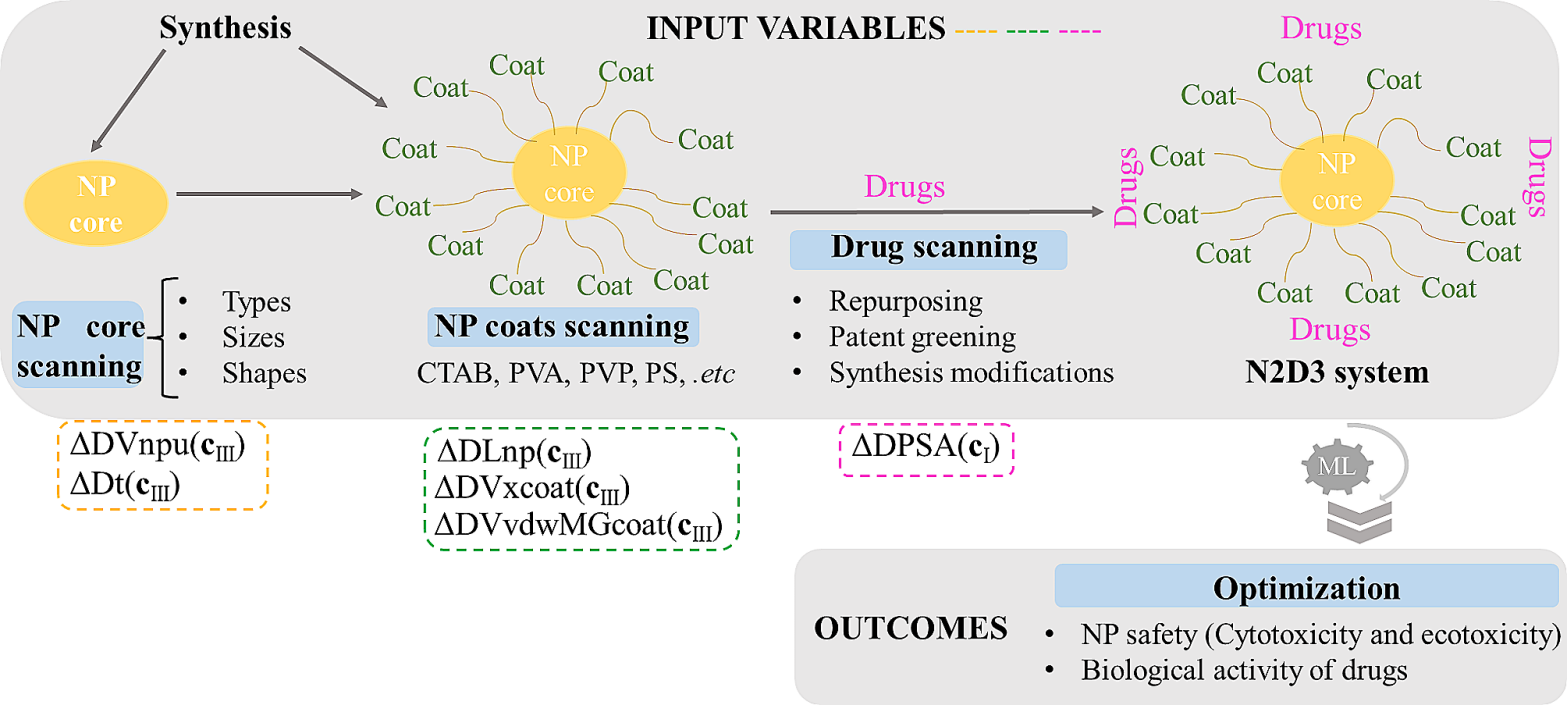
Others applications of NANO.PTML model

The NANO.PTML model has different types of applications in various stages of N2D3 system development, as shown in Fig. 10. It includes the selection of new cores, coats, or drugs. In all these cases, the N2D3 systems can be optimized in terms of drug activity and NP system safety (cytotoxicity and ecotoxicity). The first three applications involve the selection of input variables. In the NP core scanning stage, researchers can select different types, sizes, and shapes. In the NP coats scanning stage, they can select up to 16 coating agents, such as CTAB, PVA, PVP, etc. In the drug scanning stage, they can carry out NDDs synthesis modifications, repurposing, and patent greening. The synthesis modifications refer to the prediction of new N2D3 systems (different coats, cores) for new drug structures with potential NDDs activity [75]. Repurposing refers to the prediction of new NDDs for N2D3 systems from already known drugs with other activities [76]. Patent greening applications refer to the prediction of new N2D3 systems (different coats, cores) for already known NDDs [77]. In all these cases, the outcomes predicted by the NANO.PTML model can optimize NP safety and/or biological activity of NDDs. To make these predictions, we have to change the values of different input variables. In Fig. 10, we highlighted the input variables that need to be changed to make predictions for different applications. For details about the variables, see AI/ML Python Computational Models section. The variables in these four stages can be changed one by one according to the researchers’ needs; however, they can also be changed simultaneously. For example, in the simulation experiment shown in Fig. 9, we created a total of 500,000 assays in which up to 123 drugs, 53 cell lines, 16 NP coats, 5 NP shapes, and 5 NP cores were changed at the same time.

## Conclusions

The NANO.PTML model, which integrates NDDs and NP models, offers a practical solution for developing new NP system as drug carriers for neurodegenerative diseases. It effectively addresses the challenge of exploring numerous NP and NDDs compound combinations. The best-performing AI/ML model, using the DT algorithm, achieved high Sp (96.4%/96.2%) and Sn (79.3%/75.7%) in training and validation, with AUROC values of 0.97 and 0.96. Chemically synthesized Fe_3_O_4_ NPs were structurally characterized and coated with CTAB to enhance water solubility. We illustrated an example of the IFPTML-DT model application in a real experiment (reported here). To do this, we performed an experimental simulation using a large prediction dataset including 500,000 cases/empirical experiments similar to NPs studied in the experimental part. This simulation experiment showed that certain NP systems as promising candidate for further investigation, highlighting the *Lycopersicon esculentum* cell line for ecotoxicity studies according green section of Fig. 9. The MacGowan volume was significant for certain coating agents (CTAB, PS, PEG) but not for PVA. Overall, the NANO.PTML model expedites experimental research and provides reliable initial findings, reducing the reliance on time-consuming wet-lab procedures.

## Data Availability

The datasets generated and/or analyzed during the current study are available in the Figshare repository, DOI: 10.6084/m9.figshare.25450291. On the other hand, the code of the NANO.PTML models was uploaded to a GitHub repository and is available free for use by researchers. For the NANO.PTML models code the link is: https://github.com/she012/NANO.PTML-project.

## Abbreviations

AI: Artificial Intelligence refers to the use of computers to simulate intelligent behavior with minimal human involvement [78]
AUROC: Area Under Receiver Operating Characteristic is commonly used to assess the accuracy of diagnostic tests. A ROC curve that is closer to the upper left corner of the graph indicates higher test accuracy, as this point represents a sensitivity of 1 and a false positive rate of 0 (specificity of 1) [79]
BBB: Blood-Brain Barrier is a selective permeability barrier formed by the blood vessels that vascularize the central nervous system [80]
CNS: Central Nervous System is the brain and spinal cord. This system is responsible of receiving, processing, and responding to sensory information [81]
CTAB: Cetyl Trimethyl Ammonium Bromide is a cationic surfactant
DLS: Dynamic Light Scattering measures changes in scattering intensity over time caused by particles moving randomly due to Brownian motion [82]
DNDS: Drug Nanoparticle Delivery System
DRX: X Ray Diffraction has become a widely used method for examining crystal structures and atomic spacing [83]
DT: Decision Tree is a hierarchical decision support model that employs a tree-like structure to represent decisions and their potential outcomes [54]
GB: Gradient Boosting is an ensemble machine learning technique that sequentially combines the predictions of multiple weak learners, usually decision trees [57]
IF: Information Fusion is the process of integrating data from different sources
kNN: K-Nearest Neighbors is a popular machine learning technique which based on the principle that data points which are close to each other are likely to share similar labels or values [84].
LDA: Linear Discriminant Analyses is a machine learning method that aims to identify a linear combination of features that effectively distinguishes between two or more classes of objects or events [85]
NDs: Neurodegenerative Diseases
MA: Moving Average is a method used to analyze data points by calculating a series of average values from various subsets of the entire dataset [86]
MCC: Mathew’s Correlation Coefficient is a metric for binary classification that evaluates predictions by considering true positives, true negatives, false positives, and false negatives[87
ML: Machine Learning is the science of developing algorithms and statistical models that enable computer systems to perform tasks without explicit instructions, relying instead on patterns and inferences [88]
NP: Nanoparticle
NDDs: Neurodegenerative Disease Drugs
N2D3: Nanoparticle Neuronal Disease Drug Delivery systems
PMAO: Poly(Maleic Anhydride-alt-1-Octadecene)
PT: Perturbation Theory involves starting with a simple system for which a mathematical solution is already known. Then, an additional perturbation is introduced to represent a weak disturbance to the system [89]
PTOs: Perturbation Theory Operators is the linear and non-linear transformations of moving average. For example, the deviation of the moving average
RF: Radom Forest is a popular machine learning technique that combines the collective predictions of numerous decision trees to generate a combined outcome [90]
RT: Room Temperature
TEM: Transmission Electron Microscopy is a method of microscopy where a specimen is illuminated with a beam of electrons, allowing the formation of an image as the electrons pass through the specimen [91]
TGA: ThermoGravimetric Analysis is a method used to assess the thermal stability of materials, including polymers, by measuring changes in weight as a function of temperature [92]
VSM: Vibrating-Sample Magnetometer is a device used to measure the magnetic properties of a sample while it undergoes perpendicular vibrations within a uniform magnetic field [93]
